# Extensive Variation and Sub-Structuring in Lineage A mtDNA in Indian Sheep: Genetic Evidence for Domestication of Sheep in India

**DOI:** 10.1371/journal.pone.0077858

**Published:** 2013-11-11

**Authors:** Sachin Singh, Satish Kumar Jr, Atul P. Kolte, Satish Kumar

**Affiliations:** 1 CSIR-Centre for Cellular and Molecular Biology, Hyderabad, India; 2 ICAR-Central Sheep and Wool Research Institute, Avikanagar, India; 3 ICAR-National Institute of Animal Nutrition and Physiology, Bangaluru, India; BiK-F Biodiversity and Climate Research Center, Germany

## Abstract

Previous studies on mitochondrial DNA analysis of sheep from different regions of the world have revealed the presence of two major- A and B, and three minor- C, D and E maternal lineages. Lineage A is more frequent in Asia and lineage B is more abundant in regions other than Asia. We have analyzed mitochondrial DNA sequences of 330 sheep from 12 different breeds of India. Neighbor-joining analysis revealed lineage A, B and C in Indian sheep. Surprisingly, multidimensional scaling plot based on F_ST_ values of control region of mtDNA sequences showed significant breed differentiation in contrast to poor geographical structuring reported earlier in this species. The breed differentiation in Indian sheep was essentially due to variable contribution of two major lineages to different breeds, and sub- structuring of lineage A, possibly the latter resulting from genetic drift. Nucleotide diversity of this lineage was higher in Indian sheep (0.014 ± 0.007) as compared to that of sheep from other regions of the world (0.009 ± 0.005 to 0.01 ± 0.005). Reduced median network analysis of control region and cytochrome b gene sequences of Indian sheep when analyzed along with available published sequences of sheep from other regions of the world showed that several haplotypes of lineage A were exclusive to Indian sheep. Given the high nucleotide diversity in Indian sheep and the poor sharing of lineage A haplotypes between Indian and non-Indian sheep, we propose that lineage A sheep has also been domesticated in the east of Near East, possibly in Indian sub-continent. Finally, our data provide support that lineage B and additional lineage A haplotypes of sheep might have been introduced to Indian sub-continent from Near East, probably by ancient sea trade route.

## Introduction

The present day domestic livestock has been derived from taming of various wild animal species, and sub-sequently by expanding their numbers and selective breeding in captivity by man to suite his various purposes, namely; food, fiber, draught, game etc. Domestication of animals heralded a cultural and economic revolution in human history. Understanding the processes of domestication has very significant bearing not only in working out strategies for further genetic improvement and conservation of genetic variability in livestock species but also in gaining knowledge on the ancient human history including trade and culture. Molecular genetic markers, particularly, control region of maternally inherited mitochondrial genome, have been extensively used to understand the origin of different domesticated livestock species [1,2,3,4,5,6,7,8]. In certain instances, it has also been possible to identify the respective wild species that gave rise to the domestic stocks. During the last several years such studies have shown that most of the livestock species have been domesticated more than once and or in more than one geographical regions of the world as evident from the discovery of multiple and distinct mitochondrial lineages in the extant populations [6,9,10,11,12]. 

Archaeological evidence suggests that sheep might have been one of the earliest species domesticated by man ~12000 years bp in Southwestern Asia [13]. Mitochondrial DNA studies revealed the presence of two lineages- A and B in domestic sheep populations [4,14,15]. Neither of these lineages showed significant relationship with mitochondrial DNA sequences of the wild species of sheep. Subsequent studies [16,17,18,19] have demonstrated that domestic sheep has a complex domestication history involving two major (A and B) and three minor (C, D, and E) maternal lineages. Tapio and colleagues further suggested that the two major lineages were domesticated in Near East [18]. Based upon control region mtDNA sequences Hiendleder and colleagues thought that the lineage B type sheep might have originated from European mouflon (*Ovis musimon*) [4]. On the other hand Groeneveld and coworkers argued that the latter might represent the feral form of lineage B type sheep rather than being the wild originator of domestic sheep in Europe [20]. The wild ancestors of domestic sheep remain to be identified. Although mitochondrial and other DNA markers studies [21] on sheep breeds from different parts of the world have revealed very poor geographical structuring mitochondrial diversity studies have shown that lineage B is predominantly found in European regions [18,19] and lineage A is prevalent in Asia [16,22]. In a limited study, only lineage A has been reported in Indian sheep [22] (Pardeshi et al. 2007). 

Genetic studies on mitochondrial DNA have indicated that Indian sub-continent has been one of the important places of domestication of river buffalo [6,7] and zebu cattle [23]. India has a vast genetic resource of sheep diversity represented by more than 40 different recognized breeds that are distributed in various agro-climatic zones of the country [24]. Therefore, to understand the domestication of sheep, we have investigated the control region mtDNA and cytochrome b gene sequences of 330 samples from 12 different breeds from various regions of India. Analysis of our data along with those from published literature provides genetic evidence for domestication of lineage A type sheep in India and support for immigration of lineage B and additional lineage A haplotypes into India from elsewhere, probably through sea route in ancient times.

## Materials and Methods

### Ethics statement

The study involved drawing of ~ 5 ml blood from jugular vein aseptically from domestic sheep with the consent of the flock owners. There is no specific legislation for blood sample collection and hence no approval was necessary. Blood samples for all breeds, except Deccani and Nellore, were collected by Dr. Satish Kumar Jr., Ph.D. and Dr. Atul Kolte, M.V.Sc. Samples from Deccani and Nellore breeds were collected by Dr. Mahesh D. Sahare, M.V.Sc. and Dr. Avirat Swaimul, M.V.Sc. for their dissertation work. These samples were originally collected for genotyping in various research projects at CSWRI, Avikanagar.

### Sample collection

**Figure 1:**
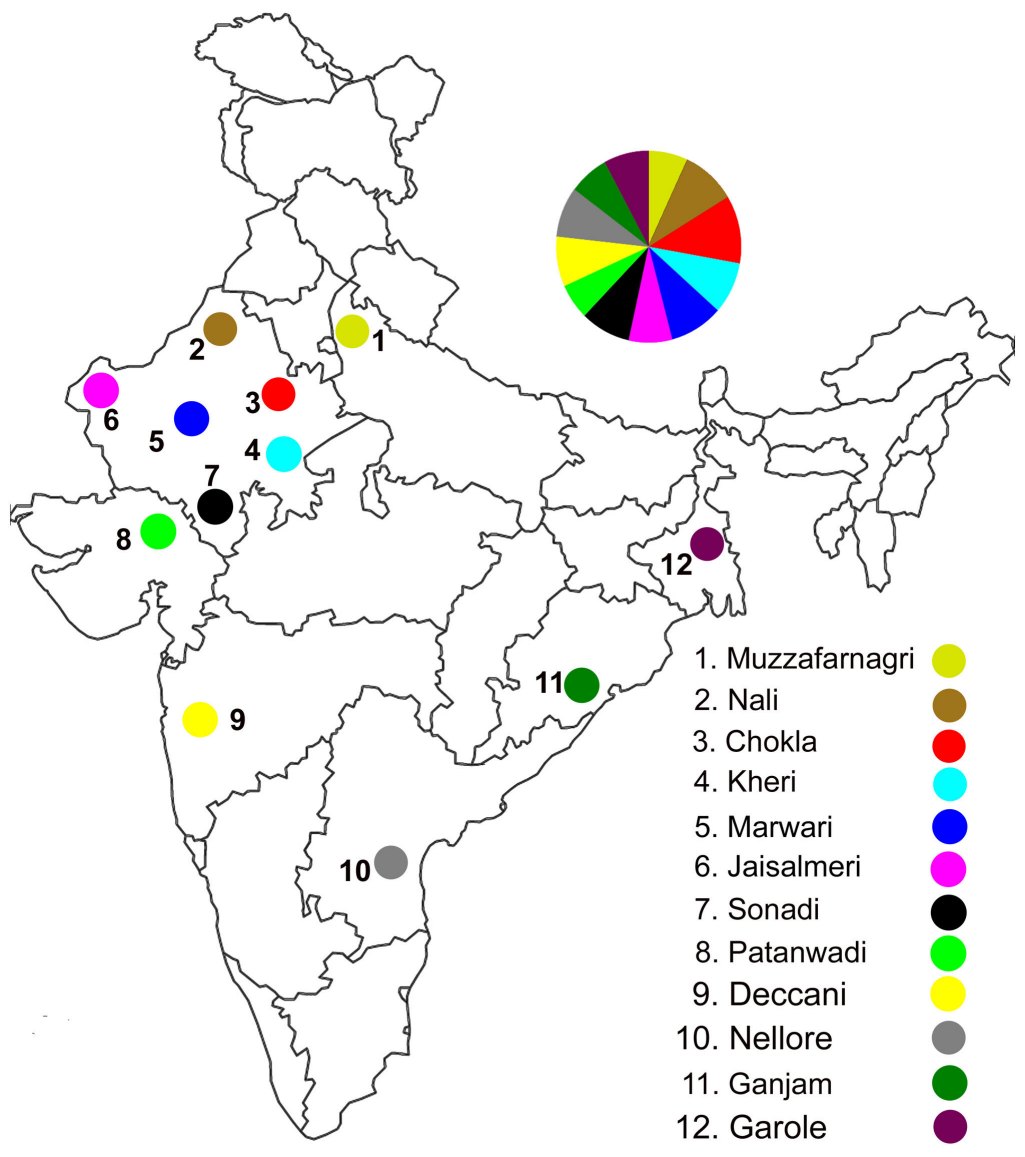
An outline map of India depicting sampling sites of Indian domestic sheep breeds used in the study. Number of animals has been shown in parenthesis against the name of the respective breed. 1- Muzzafarnagri (22); 2- Nali (31); 3- Chokla (38); 4- Kheri (29); 5- Marwari (31); 6- Jaisalmeri (25); 7- Sonadi (29); 8- Patanwadi (20); 9- Deccani (26); 10- Nellore (28); 11- Ganjam (23); 12- Garole (26).

Blood samples from 330 unrelated sheep, except those from Garole breed, representing four different agro-climatic zones of India were collected from farmers’ flocks (Figure 1). Un-relatedness of the sampled animals was ensured by covering the breeding tract of a given breed, restricting the number of samples taken from a village to one or two, and by gaining knowledge on breeding history of the selected animals based on personal interviews with flock owners. Blood samples from 26 unrelated animals of Garole breed were obtained from the Central Sheep and Wool Research Institute, Avikanagar, Rajasthan, India. DNA was isolated from jugular vein blood using organic extraction method [25].

### PCR amplification and sequencing of the mitochondrial DNA

To amplify a 721 bp of control region mtDNA fragment, Primer 3 software was used for primer designing and three overlapping sets of primers: I) forward 5’-CCAGAGAAGGAGAACAACCAA-3’ reverse 5’-CATGGTGAACAAGCTCGTGA-3’; II) forward 5’-TGTCTGTCTTAAACATGCAAACG-3’ reverse 5’-CCAAGCATCCCCAAAAATTA-3’, and III) forward 5’-GGGTATTAAACTGCTTGACCGTA-3’ reverse 5’-GCATTTTCAGTGCCTTGCTT-3’ were designed based on sheep mitochondrial reference sequence: AF010406 [26]. The coordinates of the amplicon corresponded from 15541 to 16216 nt of the reference sequence. PCR reactions were performed using 2X AmpliTaq gold PCR master Mix (Applied Biosystem, Roche Molecular System, Inc.), 5 pM each primer, 20 ng of DNA in a final volume up to 10 µl. The reactions were performed in Eppendorf thermal cycler with following conditions: initial denaturation 95°C for 5 min, followed by 30 cycles at 94°C for 1 min, 59°C for 45 sec and 72°C for 1 min and final extension at 72°C for 5 min. Single band of the PCR products were confirmed by 1.5 % agarose gel electrophoresis stained with ethidium bromide. Amplicons were treated with ExoSAP-IT as per manufactures instructions (Affymetrix). Sequencing reactions were performed using BigDye Terminator Cycle Sequencing Kit (Applied Biosystems) and the products were purified by alcohol precipitation. Purified products were analyzed in ABI 3730 automated DNA sequencer (Applied Biosystems). To amplify a 967 bp of cytochrome b gene, the following three sets of primer pairs were used: I) forward 5’-TGTCATCATCATTCTCACATGG-3’ reverse 5’-GCGATGATGAATGGGAAAATA-3’ II) forward 5’-GGACGAGGCCTATACTATGGA-3’ reverse 5’-TGAGGGGGAGTGTTAAGTGG-3’ III) forward 5’-CCTACTAATCCTCATCCTCATGC-3’ reverse 5’-GGGAGGTTGGTTGTTCTCCT-3’. PCR and sequencing reactions were setup essentially using the same condition as described above for control region mtDNA except that the annealing temperatures of 53°C for the first and third set of the primer pairs and 50°C for the second set primer pair were used. The sheep sequences were submitted to GenBank (accession numbers JX545477 - JX546133).

### Data analysis

The sequences were edited using AUTOASSEMBLER (Perkin Elmer) program and 721 bp of control region mtDNA were obtained with respect to the reference sequence. Alignment showed that control region of the majority of the sequences contained four 75 bp tandem repeats, while two sequences contained three 75 bp & one 76 bp repeats. However, three samples contained one 75 & three 76 bp tandem repeats each. For comparative analysis of our sequences, control region mtDNA sequences of domestic and wild sheep were collated from NCBI as follows: (DQ242050 - DQ242455) [18], (DQ491576 - DQ491736) [27], (EU019130 - EU019189), (AY829376 - AY829430) [16], (DQ903228 - DQ903304) [28], (DQ852280 - DQ852289) [19], *Ovis ammon ammon* AF242347 [4], *Ovis vignei arkal* AY091489 [4], *Ovis ammon nigrimontana* AY091494 [4], *Ovis vignei bochariensis* AY091491 [15], *Ovis ammon collium* AY091492 [4] and *Ovis Vignei arkal* AY091489 [4]. The cytochrome b gene sequences of 326 sheep from the present study were analyzed along with following sequences available from the data bases: (FJ218019 - FJ218150) [22], (DQ903208 - DQ903227), (AY879464 - AY879584) [29], (DQ097407 - DQ097430) [17] (DQ851886 - DQ852082) [19]. In order to avoid tandem repeats present in sheep control region mtDNA, we considered 432 bp comprising two fragment (I) 103 bp spanning from 15541 to 15643 and (II) 329 bp from 15933 to 16261 with respect to the reference sequence.

ClustalX version 65534.0.10.0 program [30] was used for the alignment of sequences. GeneDoc version 2.7.0.0 package was used for formatting the aligned sequences to make them compatible for the desired software. MEGA 5 version 5.0.1.102 [31] was used to construct Neighbor-joining tree using Tamura-Nei model [32] with 10,000 replicates. Maximum Likelihood tree was also constructed with Hasegawa-Kishino-Yano (HKY) [33] model (*+G* = 0.37, I = 0.62) with 5 categories. DNA alignment software version 1.3.1.1 (www.fluxus-engineering.com) was used to convert the aligned sequence into RDF binary format. Reduced median network [34,35,36] was drawn using Network software version 4.6.1.0 (www.fluxus-engineering.com) with parameters set to a weight of 2 and threshold value of 1. Haplotype, nucleotide diversity and its standard error, Fu’s Fs statistics [37], mismatch distribution [38], AMOVA (analysis of molecular variance), and the population pairwise differences (F_ST_) values were calculated using Arlequin version 3.5.1.2 software [39]. Pairwise F_ST_ values were calculated with 10,000 bootstrap and values were displayed as a multidimensional scaling (MDS) plot with a stress value = 0.12 using SPSS 11.0 software.

## Results

### Genetic variation within control region mtDNA sequences

**Figure 2:**
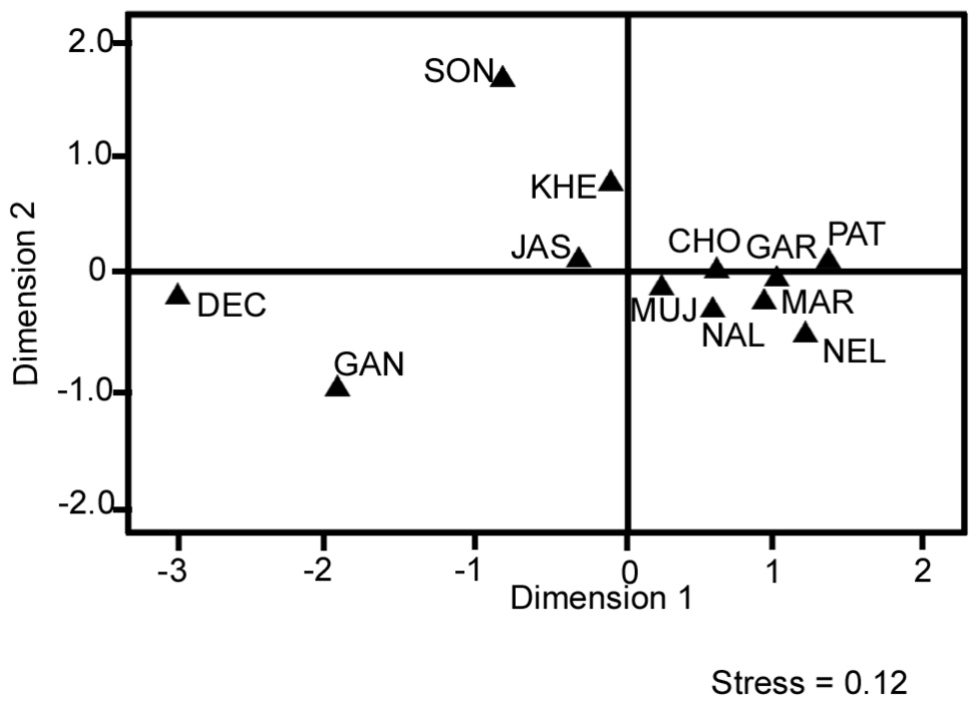
Multidimensional scaling (MDS) plot using pairwise F_ST_ values between Indian sheep breeds. MUJ- Muzzafarnagri; NAL- Nali; CHO- Chokla; KHE- Kheri; MAR- Marwari; JAS- Jaisalmeri; SON- Sonadi; PAT- Patanwadi; DEC- Deccani; NEL- Nellore; GAN- Ganjam; GAR- Garole.

We aligned 432 bp of control region mtDNA sequences of 330 samples of Indian sheep belonging to 12 different breeds from four agro-climatic regions of India (Figure 1). The sample size for a given breed ranged from 20 to 38. Multiple sequence alignment revealed the presence of 77 nucleotide variable sites, out of which 64 were parsimony informative and 13 were singleton sites. The comparison of 330 sequences showed 193 mitochondrial haplotypes; among them 146 haplotypes were unique. The most frequent haplotype was observed in 30 individuals from seven breeds. The number of haplotypes for a given breed ranged from 14 to 30 and the haplotype diversity values ranged from 0.916 ± 0.05 (Patanwadi) to 0.993 ± 0.01 (Jaisalmeri) and the nucleotide diversity varied from 0.015 ± 0.008 (Deccani) to 0.036 ± 0.018 (Sonadi) [Table 1]. The high transition: transversion ratio (18.24: 1) revealed strong bias towards transition in Indian domestic sheep as has been reported for various species [5,7]. The breed effect on mitochondrial genetic variation was analyzed by AMOVA. The breed component contributed 13.41% to the total mitochondrial genetic variation (P < 0.01) indicating significant breed differentiation with respect to maternal lineages of Indian sheep. To further understand the genetic differentiation among breeds, F_ST_ values were calculated between all breed pairs based on the nucleotide differences. Maximum differentiation was observed between Deccani and Patanwadi (44.90%), while no significant differentiation was observed among a group of breeds such as Marwari, Garole, Chokla, Nali, Patanwadi, Muzzafarnagri and Nellore. Calculated pairwise F_ST_ values were also analyzed by multidimensional scaling (MDS) plot with stress value of 0.12 (Figure 2). MDS plot revealed three major groups: 1) Deccani and Ganjam; 2) Chokla, Marwari, Muzzafarnagri, Nali, Nellore, Patanwadi, Jaisalmeri, Garole and Kheri; 3) Sonadi. Consistent with these results AMOVA analysis after classification of Deccani and Ganjam breeds as one group versus the remaining breeds as a single group explained 21.15% (P < 0.01) of total genetic variation. Similarly, when Sonadi breed was treated as an additional group 23.33 % (P < 0.01) of the total genetic variation was accounted by this grouping.

**Table 1:** Haplotype and nucleotide diversity, and relative contribution of various mtDNA lineages in Indian sheep breeds.

Sr.No.	Breed	Number of samples	Number of haplotypes	Haplotype diversity	Nucleotide diversity	Relative contribution of various lineages
1	Muzzafarnagri	22	18	0.980 ± 0.021	0.022 ± 0.012	A =0.83; B=0.16
2	Nali	31	26	0.989 ± 0.011	0.021 ± 0.011	A=0.87; B=0.10; C=0.03
3	Chokla	38	30	0.980 ± 0.013	0.022 ± 0.012	A=0.85; B=0.15
4	Kheri	29	26	0.993 ± 0.011	0.031 ± 0.016	A=0.66; B=0.34
5	Marwari	31	21	0.918 ± 0.043	0.018 ± 0.010	A=0.88 ; B=0.12
6	Jaisalmeri	25	23	0.993 ± 0.013	0.030 ± 0.016	A=0.80; B=0.12; C=0.08
7	Sonadi	29	21	0.974 ± 0.015	0.036 ± 0.018	A=0.52; B=0.48
8	Patanwadi	20	14	0.916 ± 0.055	0.015 ± 0.008	A=0.90; B=0.10
9	Deccani	28	23	0.979 ± 0.018	0.015 ± 0.008	A=1.0
10	Nellore	28	25	0.992 ± 0.012	0.015 ± 0.008	A=0.93; B=0.07
11	Ganjam	23	19	0.980 ± 0.020	0.016 ± 0.009	A=1.0
12	Garole	26	14	0.920 ± 0.034	0.020 ± 0.011	A=0.88; B=0.12
		330	193	0.987 ± 0.003	0.025± 0.013	A=0.84; B=0.15; C=0.009

### Phylogenetic analysis

**Figure 3:**
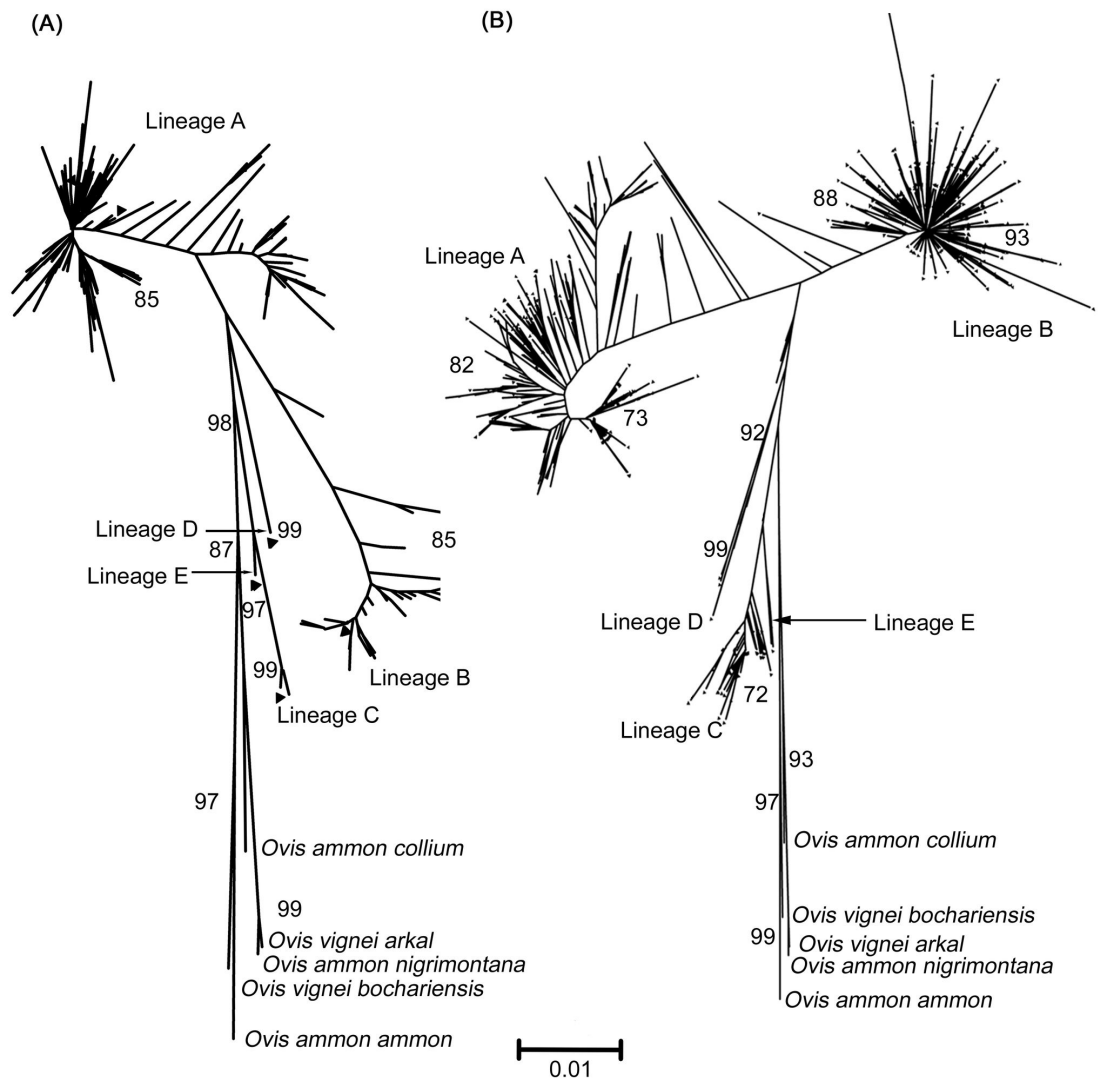
Neighbor-joining tree of domestic sheep based on 432 bp of control region mtDNA. (A) Neighbor-joining tree of mtDNA sequences of Indian sheep (330) along with representative samples of five lineages (▲), namely; A, B, C, D & E. Indian sheep show three lineages, namely; A, B and C. (B) Neighbor-joining tree of mtDNA sequences of the Indian (330), Chinese (129), Central Asian, Caucasian and European (406), Portuguese (161), and West Balkan (60), sheep along with representative samples of five lineages (▲), namely; A, B, C, D & E . The sequences of wild *Ovis* species have been used as outgroups. MEGA 5 version 5.0.1.102 was used to construct the trees using Tamura-Nei model with 10,000 bootstrap. Numbers above a given branch represent bootstrap support for the branch as a percentage out of 10,000 re samplings.

Neighbor-joining tree based on 432 bp of control region mtDNA of 330 sheep samples from India and rooted with wild sheep sequences showed the presence of three distinct lineages, namely; A, B and C, out of the five lineages reported in this species thus far (Figure 3A). Lineage A was predominant in Indian sheep (84%) as compared to lineage B (15%) [Table 1]. Lineage C was found in less than 1% of the animals analyzed. Maximum likelihood tree also showed a similar topology (Figure S1). Interestingly, the sequence diversity in lineage A of Indian sheep as compared to that of sheep breeds from other parts of the world was high (Figure 3B). Indeed, several branches of lineage A in the tree were exclusive to Indian sheep indicating sub - structuring of the lineage A. The relative contribution of lineage A and B to different breeds was extremely variable (Table 1). While majority of the breeds had predominantly lineage A (66 to 93%), Deccani and Ganjam breeds had only lineage A while in Sonadi breed the two lineages were almost equally represented.

### Reduced median network analysis

**Figure 4:**
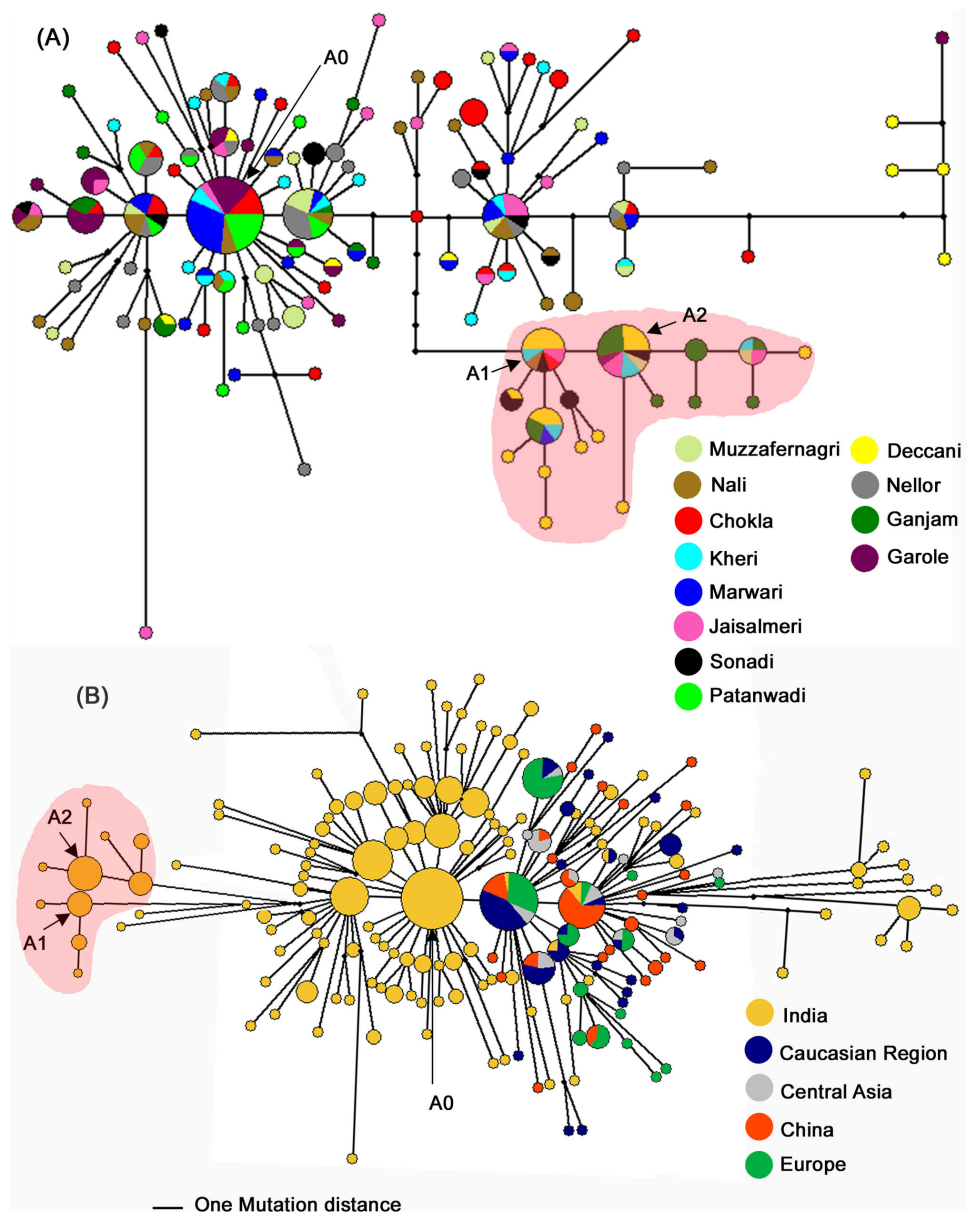
A reduced median network of lineage A type of domestic sheep based on 432 bp of control region mtDNA. (A) Network based on sequences of Indian sheep (279). (B) Network based on Indian (279), Chinese (46), Central Asian, Caucasian and European (98), Portuguese (6) and West Balkan (4) sheep sequences. The nodes represent haplotypes and the size of a given node is proportional to the frequency of the samples represented in that node. Branch length is proportional to the mutational differences between. The smallest node represents one individual animal.

Given the complexity of lineage A in Indian sheep as revealed by phylogenetic analysis (Figure 3) we investigated the relationships among various haplotypes of 279 animals of lineage A and their distribution in various breeds (Figure 4A). Reduced median network analysis (Figure 4A) showed multiple expanding haplotypes and several singletons radiating from these expanding haplotypes indicating the expansion of lineage A type sheep populations in India. Although there was a main expanding haplotype there were many additional haplotypes showing expansion both a few mutation steps away from the main haplotype as well as ten mutation steps away from the main haplotype. The main haplotype was found in seven of the twelve breeds studied. Differential contribution of various breeds to different haplotypes was evident. Most significantly, Deccani and Ganjam breeds had predominantly a cluster of haplotypes which were isolated from the main expanding haplotypes (shaded area; Figure 4A). When all Indian lineage A type sequences were grouped in two categories i.e. isolated haplotypes (shaded area; Figure 4A) and the remaining sequences AMOVA analysis showed that 66.48 % of the total variation was explained by this classification confirming the divergence of the isolated haplotypes from the remaining haplotypes of Indian sheep. 

To investigate the relationship of lineage A type haplotypes of Indian sheep with those of other regions of the world, we performed reduced median network analysis of 433 sequences including 279 Indian sequences (Figure 4B). The network showed three major radiating nodes at single mutation step representing 19, 28 and 36 samples each, respectively. The first two smaller radiating nodes contained samples from all geographical regions while the largest node comprised of only Indian samples. Interestingly, out of a total of 172 haplotypes 118 were India specific and four Indian haplotypes were shared with those of other regions of the world indicating extreme diversity and isolation of Indian lineage A type haplotypes. Consistent with these observations the nucleotide diversity of lineage A in Indian sheep was 0.014 ± 0.007 in comparison to this value ranging from 0.009 ± 0.005 to 0.01 ± 0.005 in other regions.

### Demography of lineage A type sheep

**Figure 5:**
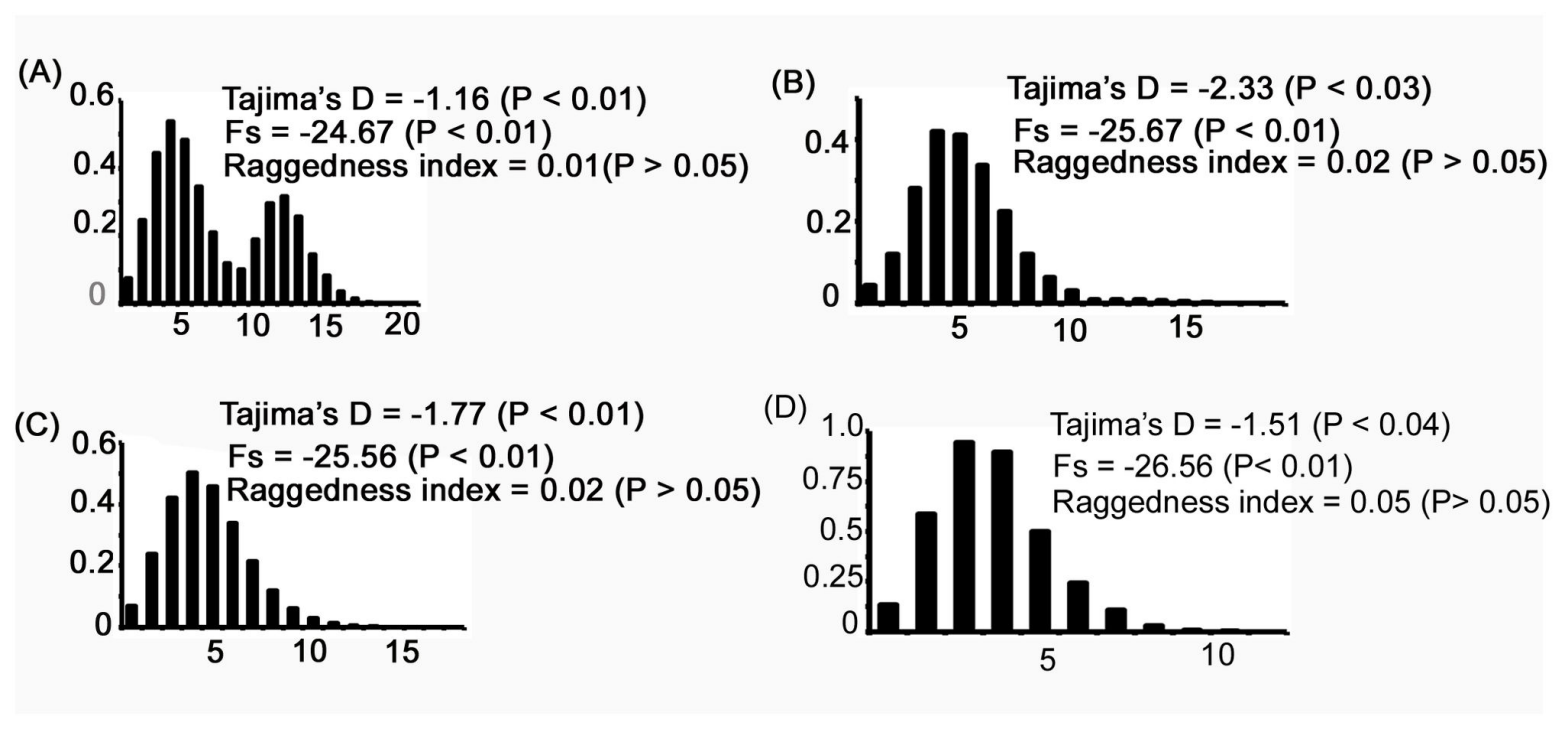
Mismatch analysis based on 432 bp of control region mtDNA sequences of lineage A sheep from different geographical regions of the world. A) Distribution curve of lineage A sequences from India (279). B) Distribution curve of China (46), Central Asia, Caucasian and Europe (98), Portugal (6), and West Balkan (4). C) Distribution curve of 223 lineage A sequences from India, after exclusion of 56 samples present in isolated haplotypes (shaded area; Figure 4A). D) Distribution curve of 56 samples of isolated haplotypes (shaded area; Figure 4A).

 Pairwise mismatch distribution of mitochondrial sequences has been extensively used to understand the demographic history of different populations [40]. Unimodal distribution is taken as an evidence for population expansion while ragged multimodal distribution is indicative of constant population size. Further, heterogeneity of domestication events on account of time of domestication and or differences in the founding mitochondrial lineages have also been inferred from mismatch distribution of mitochondrial sequences [17,19]. We obtained bimodal distribution curve (raggedness = 0.01) of Indian lineage A type sequences with maxima around 4 to 5 mutation differences between a given pair of sequences along with a additional smaller peak around 12 mutation differences (Figure 5A). Distribution curve was derived from the lineage A type sequences from European, Caucasian, Central Asian and Chinese region and a smooth unimodal curve was obtained. It may be recalled that the reduced median network analysis showed isolated clusters of haplotypes around 10 mutation steps away from the major expanding haplotypes in Indian sheep (shaded area Figure 4A), and therefore, it was possible that the secondary peak from Indian samples resulted from the inclusion of isolated clusters of haplotypes (shaded area Figure 4A). Indeed, when the latter haplotypes were excluded from Indian samples a smooth unimodal mismatch distribution was obtained (Figure 5C). These results indicated that Indian type A sequences and those from other regions of the world had similar demographic history. However distribution curve (raggedness = 0.05) (Figure 5D) of isolated cluster of haplotypes (shaded area; Figure 4A) showed maxima around 3 mutation differences between a given pair of haplotypes.

##  Discussion

Control region mtDNA analysis [18,19,27] and genome wide SNP studies [21] have revealed extensive variability in the extant genetic base of domestic sheep indicating contributions from very diverse wild ancestral stocks in the process of domestication. Kijas and coworkers investigated the relative suitability of various components of mitochondrial genome to establish the relationship among domestic and wild sheep and found that the results obtained with control region were consistent with those from whole mitochondrial genome [41]. Using control region of mtDNA, five maternal lineages, namely; A, B, C, D and E have been reported to have contributed in the process of domestication of this species. Lineage B has close relationship with European mouflon, *Ovis musimon* [4] while wild ancestors of other lineages are not yet established. Out of the two major lineages, lineage B is predominant in sheep from Middle East, Caucasian and Europe [18,19,27], while in Chinese sheep lineage A is prevalent [12,16]. A limited study on Indian sheep has found only lineage A [22]. In the present study, we analyzed 330 animals from 12 recognized sheep breeds from different parts of India, particularly sampling a large number of breeds from northwestern region of the country, the latter representing significant areas of the erstwhile Indus valley civilization. Out of the five known maternal lineages, we found three lineages, namely; A, B and C in India (Figure 3 and Figure S2A). Lineage A was present in 84% of the animals studied, confirming the previous results that lineage A was predominant in Indian sheep [22]. Distribution of haplotypes in various sheep breeds has revealed a very week population structure with respect to the geography [12,29]. Surprisingly, in our study multidimensional scaling plot of F_ST_ values (Figure 2) and AMOVA analysis differentiated 12 breeds in three major groups, namely; 1) Deccani and Ganjam, 2) Sonadi, and 3) the remaining nine breeds as one cluster, albeit without any apparent geographical indications. A closer examination of mitochondrial sequences of Sonadi, Deccani and Ganjam breeds from the Northwestern, Westcentral and Eastern parts of India, respectively revealed that these breeds differed significantly with respect to the relative contribution of lineage A and B as compared to the remaining Indian breeds (Table 1). While Deccani and Ganjam breed had exclusively lineage A Sonadi animals had these two lineages in almost equal proportion, in contrast to 66% to 93% contribution of lineage A in the remaining breeds. Further, it was noteworthy that the majority of Deccani and Ganjam animals did not have the most frequent haplotypes of lineage A of Indian sheep (Figure 4A). Indeed, Deccani and Ganjam animals shared among themselves a large number of haplotypes isolated from the main expanding haplotype of lineage A of Indian sheep (shaded area; Figure 4A).

Most interestingly, the survey of mitochondrial sequences of Indian breeds and their comparison with those from other regions of the world showed extremely high genetic diversity in lineage A (Figure 4B) in contrast to lineage B in Indian sheep (Figure S3). In neighbor-joining tree and reduced median network of lineage A there were several branches and haplotypes that were exclusive to Indian sheep. On the other hand, Indian animals were present all over the network (Figure 4B). These conclusions were also supported by reduced median network analysis using cytochrome b gene sequences (Figure S2B). Although it has been suggested that the lineage A type sheep might have been domesticated in Near East [18] no wild ancestor of lineage A has been identified thus far and specifically there is no evidence in support of Argali and Urial being the originators of the modern day domestic sheep [15] & [41]. It is fairly assumed that the centers of domestication would have higher genetic variability and as one moves away from such centers the genetic variability would drop [42,43]. In this context, it may be noted that on the basis of high genetic diversity in Chinese sheep [12] it has been suggested that sheep might have been independently domesticated in regions other than Near East. Our study revealed that Indian sheep has comparatively high mitochondrial nucleotide diversity. In reduced median network (Figure 4B) the most frequent node and several secondary expanding haplotypes were exclusively composed of Indian animals. The poor haplotype sharing of Indian and non-Indian lineage A is a strong genetic evidence in support of multiple domestication of this lineage. Our data taken together with the previously published results support the following scenario of the sheep domestication. We propose that the present day lineage A sheep was also domesticated East of Near East. The genetic evidence in the present study indicates possible domestication of sheep in Indian sub-continent from wild animals having lineage A haplotypes different from those of non-Indian sheep. It may be noted that in reduced median network some of Indian animals appeared to segregate at the fringes of the network (shaded area; Figure 4A). We cannot rule out whether the isolated clusters of haplotypes in Indian samples may indicate secondary expansion of some of the haplotypes at the periphery of the network after the initial domestication event (shaded area; Figure 4A)) of lineage A in India or these isolated haplotypes reflect yet another independent domestication event of lineage A in Indian sub-continent. The preferential representation of Deccani and Ganjam breeds in the isolated cluster of haplotypes (shaded area; Figure 4A) might be the result of secondary expansion of these haplotypes in the regions represented by these breeds. Templeton and coworkers [44] have argued that in gradually expanding population some haplotypes on the margins of the network may expand disproportionally and spread to new areas. In this context it is interesting to note that although there is a relatively high contribution of Deccani and Ganjam breeds to these isolated haplotypes other breeds are also represented in these haplotypes suggesting that expansion of these haplotypes might have predated the differentiation of Deccani and Ganjam breeds from the remaining Indian breeds. However, the differential demographic history of the main Indian lineage A and the subset of Indian lineage A type represented by these isolated haplotypes (Figure 5B and D) may also argue in favor of the latter haplotypes representing an additional independent domestication event. Although we have provided a strong genetic evidence for domestication of lineage A in Indian sub-continent neither a suitable wild ancestor has been identified nor there any archeological evidence to support the domestication of sheep in Indian sub-continent. Further mismatch distribution showed similar demographic history of Indian and non-Indian lineage A sheep (Figure 5B and Figure 5C). 

 Comparison of Indian lineage A and Chinese lineage A sheep with European sheep showed that Chinese lineage A was more similar to European sheep than to Indian sheep (Figure 4B). However, in spite of this similarity the major haplotype in Chinese animals was a minor haplotype in European sheep. Notwithstanding poor sharing of lineage A haplotypes of Indian and Chinese sheep as result of the presence of several exclusive haplotypes in Indian animals, the recent SNP data indicate otherwise that the sheep from these two regions are similar. One of the possible explanations could be that Chinese sheep represent a maternal lineage more akin to European sheep but with male mediated introgression from Indian sheep. SNP genotyping and large scale sequencing of Indian and Chinese sheep would be necessary to understand this apparent paradox. Generally, it is agreed that domestication of lineage B type sheep has taken place in Near East [18]. Our data on lineage B of Indian sheep, particularly the complete absence of this lineage from a few Indian breeds from West-central and Eastern parts of the country ( [16] & present study) are consistent with the arrival of lineage B from outside of Indian sub-continent. Interestingly, the relative contribution of lineage B to various breeds is extremely variable (0% to 48%), the highest being in Sonadi breed (Table 1 & Figure S4). The present-day breeding tract of Sonadi breed were parts of the contemporary settlements of the suggested Harappan domain of the Indus civilization and not very far away from the very important ancient Lothal port of Indus civilization [45]. This port had important trading links with the far corners of West Asia and Africa during the times of Indus civilization. The high frequency of lineage B in this region in India may be interpreted as a circumstantial evidence in support of this port being one of the important entry points of this lineage into Indian sub-continent. As one moves away from this region, the contribution of lineage B in various Indian breeds decreases (Figure S4). More interestingly, the same scenario continues in the Pakistani sheep i.e. lineage B is present only in 16 % of these animals (Babar, personal communication).

In conclusion, we have provide strong genetic evidence of domestication of the present day lineage A type sheep in east of Near East, possibly in Indian sub-continent. In contrast to the poor phylogeographic structuring of mitochondrial genetic diversity in sheep from different regions of the world we found strong breed differentiation in Indian sheep as a consequences of genetic drift in lineage A or founding effects due to an additional domestication event in the regions of the present day Decanni or Ganjam breeds and differential contribution of lineage B to various Indian breeds. Further, our data provide support that lineage B type sheep and additional lineage A haplotypes of sheep would have been introduced to Indian sub-continent from Near East, probably by ancient sea trade route. Lately, genetic evidence has been accumulating to suggest that cattle and buffalo were domesticated in South Asia [6,7,23,46]. Our study on Indian sheep adds one more species to the list of the animals that possibly might have been domesticated in Indian sub-continent.

## Supporting Information

Figure S1 **Maximum likelihood tree of Indian domestic sheep sequences (330) based on 432 bp of control region mtDNA sequences along with representative samples of five lineages (▲), namely; A, B, C, D & E.** Analysis revealed presence of three distinct lineages, namely; A, B and C out of five lineages reported so far. The sequences of wild *Ovis* species have been used as outgroups. MEGA 5 version 5.0.1.102 was used to construct the trees with 10,000 bootstrap. Numbers above a given branch represent bootstrap support for the branch as a percentage out of 10,000 re samplings.
(TIF)

Figure S2 **A reduced median network analysis based on 967 bp of cytochrome b gene.** (A) Reduced median network of 326 Indian domestic sheep sequences of the present study showed three distinct lineages, namely; A, B and C out of five lineage reported so far. (B) Reduced median network of 824 sequences of sheep from different region of the world revealed extensive haplotype diversity of lineage A in Indian sheep. The nodes represent haplotypes and the size of a given node is proportional to the frequency of the samples represented in that node. Branch length is proportional to the mutational differences between nodes. The smallest node represents one individual animal.
(TIF)

Figure S3 **A reduced median network analysis based on 432 bp of control region mtDNA of lineage B sheep found in different geographical regions of the world.** Network comprised Indian (48), Chinese (47), Central Asian (21), Caucasian (94), European (380) sheep sequences. The nodes represent haplotypes and the size of a given node is proportional to the frequency of the samples represented in that node. Branch length is proportional to the mutational differences between nodes. The smallest node represents one individual animal.
(TIF)

Figure S4 **Geographical map of India depicting relative distribution of Lineage A, B and C in different Indian sheep breeds used in this study.**
(TIF)
