# Supplementary figures and images for: Extensive Variation and Sub-Structuring in Lineage A mtDNA in Indian Sheep: Genetic Evidence for Domestication of Sheep in India
Source: PLoS One. 2013 Nov 11;8(11):e77858. doi: 10.1371/journal.pone.0077858 (PMC3823876; doi:10.1371/journal.pone.0077858)

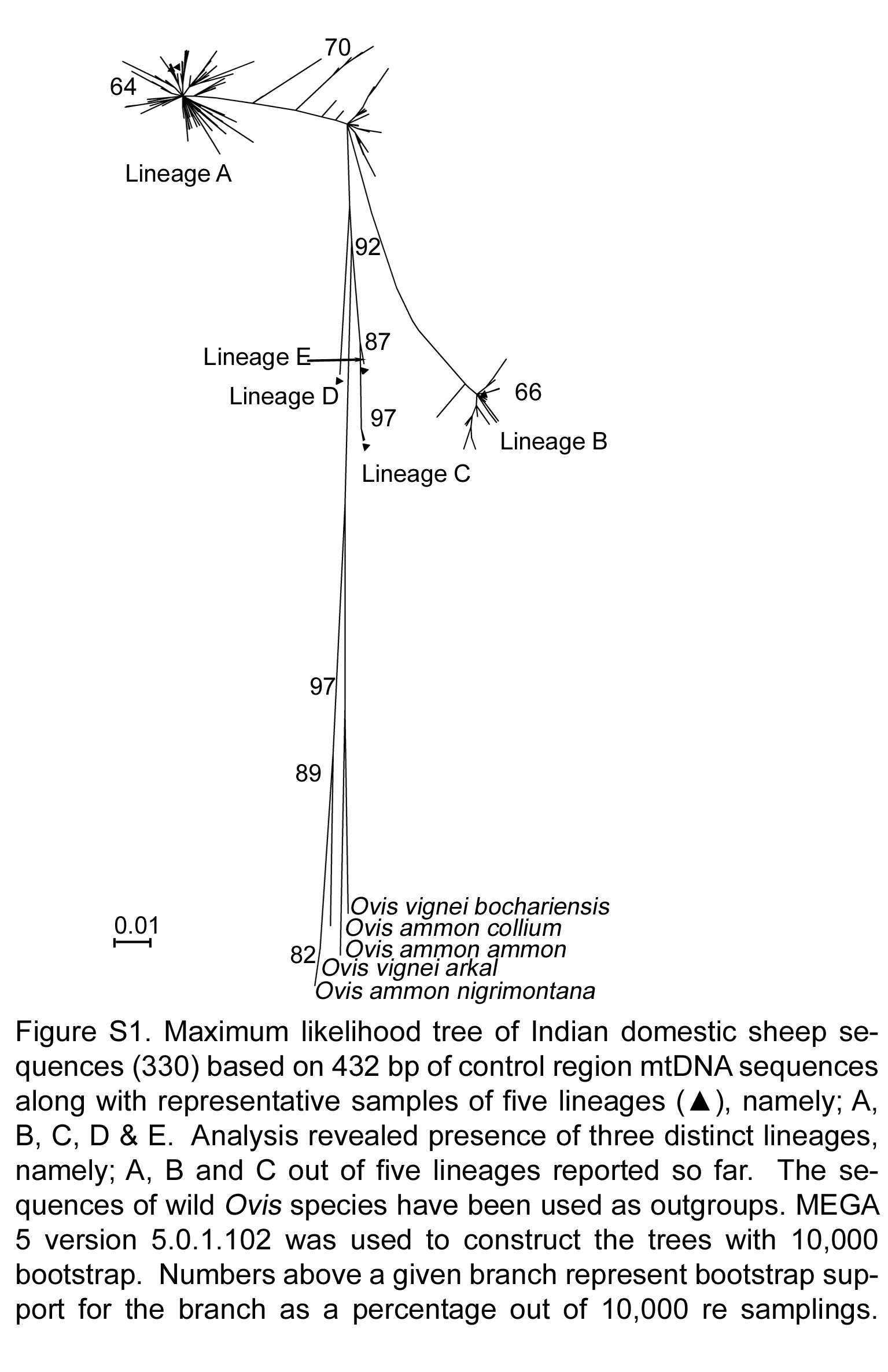

Supplement: Figure S1 — Maximum likelihood tree of Indian domestic sheep sequences (330) based on 432 bp of control region mtDNA sequences along with representative samples of five lineages (▲), namely; A, B, C, D & E. Analysis revealed presence of three distinct lineages, namely; A, B and C out of five lineages reported so far. The sequences of wild Ovis species have been used as outgroups. MEGA 5 version 5.0.1.102 was used to construct the trees with 10,000 bootstrap. Numbers above a given branch represent bootstrap support for the branch as a percentage out of 10,000 re samplings. (TIF) [file pone.0077858.s001.tif]

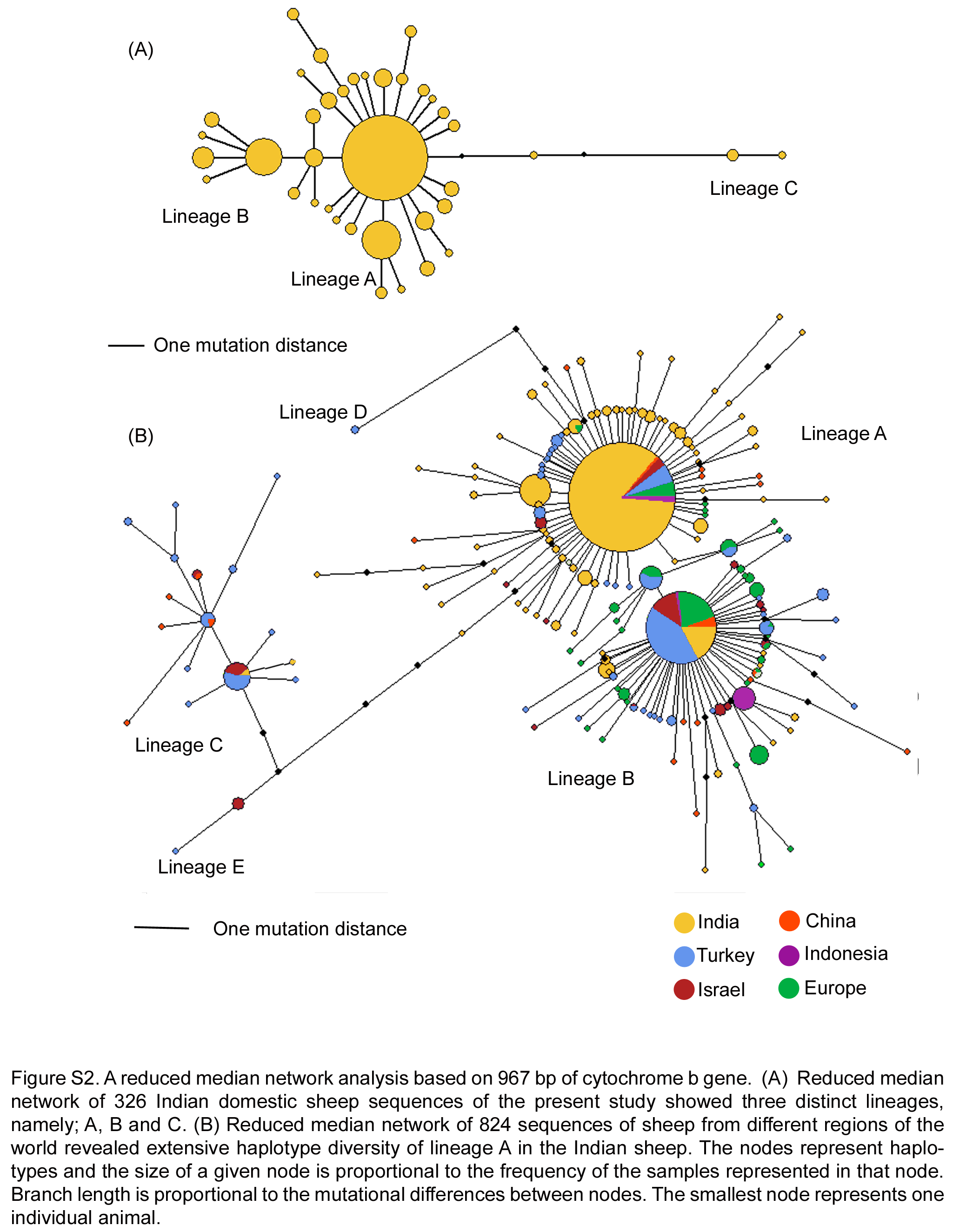

Supplement: Figure S2 — A reduced median network analysis based on 967 bp of cytochrome b gene. (A) Reduced median network of 326 Indian domestic sheep sequences of the present study showed three distinct lineages, namely; A, B and C out of five lineage reported so far. (B) Reduced median network of 824 sequences of sheep from different region of the world revealed extensive haplotype diversity of lineage A in Indian sheep. The nodes represent haplotypes and the size of a given node is proportional to the frequency of the samples represented in that node. Branch length is proportional to the mutational differences between nodes. The smallest node represents one individual animal. (TIF) [file pone.0077858.s002.tif]

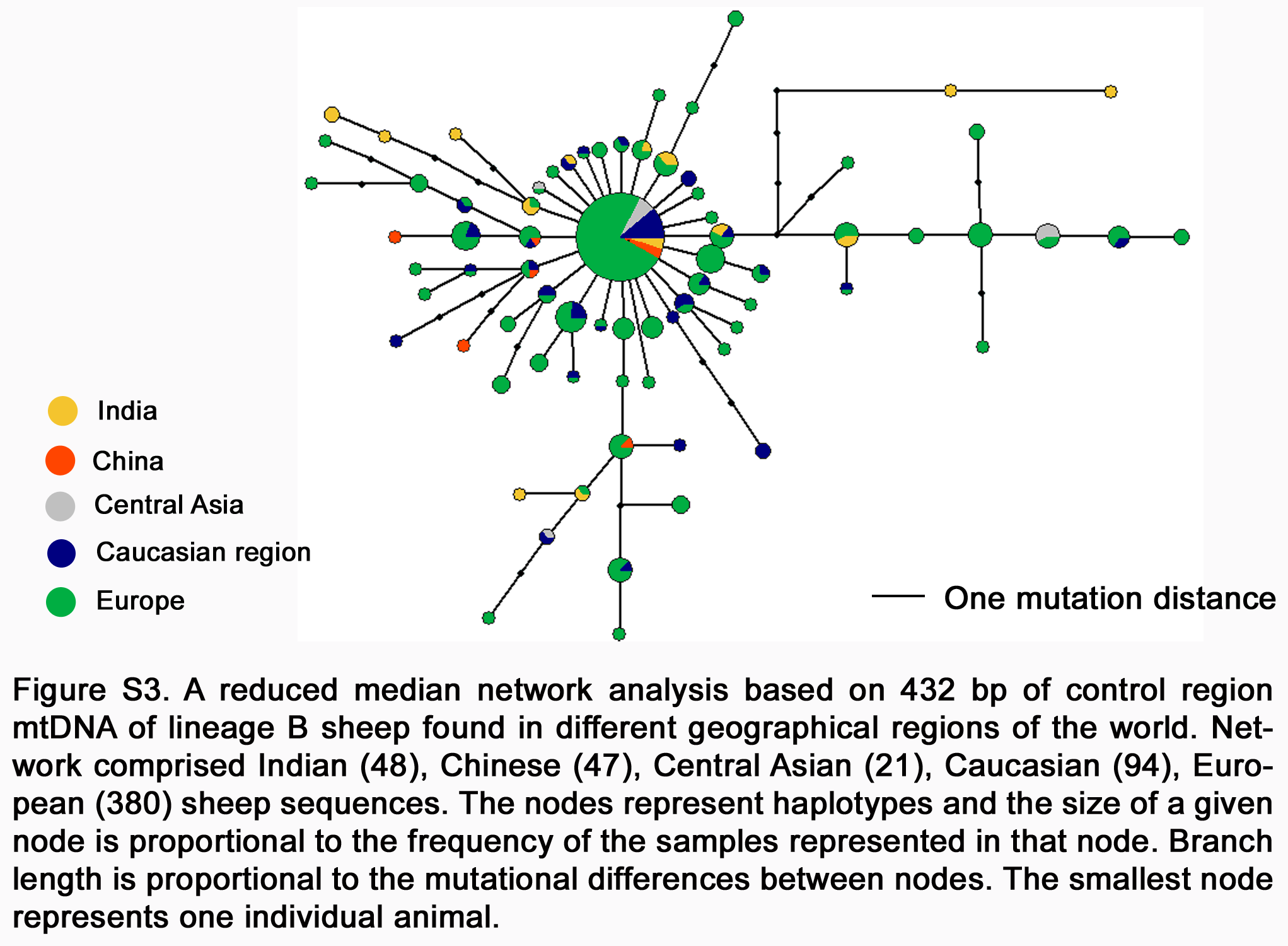

Supplement: Figure S3 — A reduced median network analysis based on 432 bp of control region mtDNA of lineage B sheep found in different geographical regions of the world. Network comprised Indian (48), Chinese (47), Central Asian (21), Caucasian (94), European (380) sheep sequences. The nodes represent haplotypes and the size of a given node is proportional to the frequency of the samples represented in that node. Branch length is proportional to the mutational differences between nodes. The smallest node represents one individual animal. (TIF) [file pone.0077858.s003.tif]

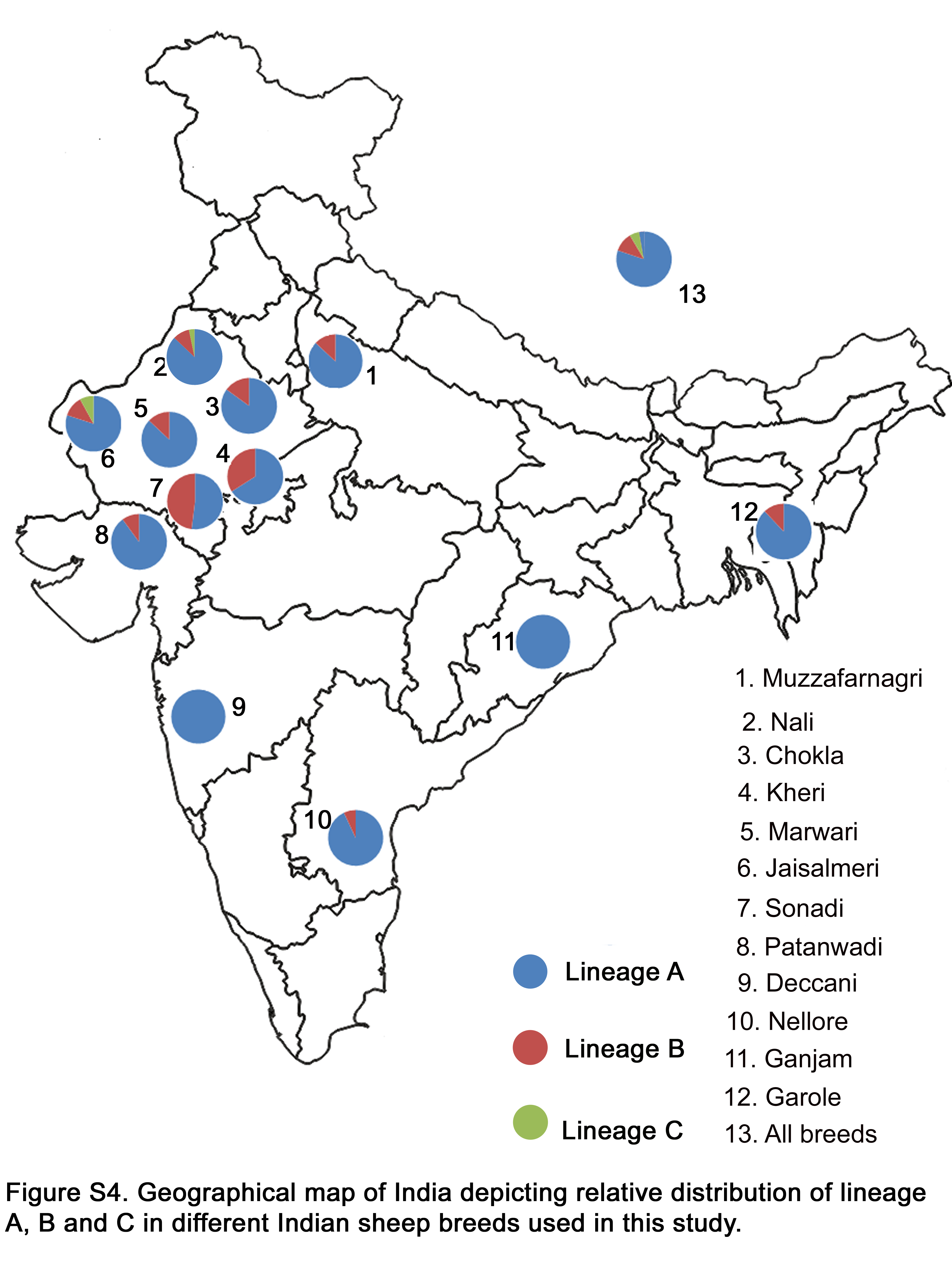

Supplement: Figure S4 — Geographical map of India depicting relative distribution of Lineage A, B and C in different Indian sheep breeds used in this study. (TIF) [file pone.0077858.s004.tif]
